# A Direct Method to Extract Transient Sub-Gap Density of State (DOS) Based on Dual Gate Pulse Spectroscopy

**DOI:** 10.1038/srep24096

**Published:** 2016-06-14

**Authors:** Mingzhi Dai, Karim Khan, Shengnan Zhang, Kemin Jiang, Xingye Zhang, Weiliang Wang, Lingyan Liang, Hongtao Cao, Pengjun Wang, Peng Wang, Lijing Miao, Haiming Qin, Jun Jiang, Lixin Xue, Junhao Chu

**Affiliations:** 1 Ningbo Institute of Materials Technology and Engineering, Chinese Academy of Sciences, Ningbo, 315201, China; 2 Department of Information Science and Electronic Engineering, Zhejiang University, Hangzhou, Zhejiang, 310027, China; 3 Institute of Circuits and Systems, Ningbo University, Ningbo 315211, China; 4 National Laboratory for Infrared Physics, Shanghai Institute of Technical Physics, Chinese Academy of Sciences, Shanghai 200083, China; 5 Laboratory of Polar Materials and Devices, Ministry of Education,East China Normal University, 500 Dong-chuan Road, Shanghai 200241, China

## Abstract

Sub-gap density of states (DOS) is a key parameter to impact the electrical characteristics of semiconductor materials-based transistors in integrated circuits. Previously, spectroscopy methodologies for DOS extractions include the static methods, temperature dependent spectroscopy and photonic spectroscopy. However, they might involve lots of assumptions, calculations, temperature or optical impacts into the intrinsic distribution of DOS along the bandgap of the materials. A direct and simpler method is developed to extract the DOS distribution from amorphous oxide-based thin-film transistors (TFTs) based on Dual gate pulse spectroscopy (GPS), introducing less extrinsic factors such as temperature and laborious numerical mathematical analysis than conventional methods. From this direct measurement, the sub-gap DOS distribution shows a peak value on the band-gap edge and in the order of 10^17^–10^21^/(cm^3^·eV), which is consistent with the previous results. The results could be described with the model involving both Gaussian and exponential components. This tool is useful as a diagnostics for the electrical properties of oxide materials and this study will benefit their modeling and improvement of the electrical properties and thus broaden their applications.

Amorphous oxide semiconductors are widely used semiconductor materials for thin-film transistors (TFTs) in modern integrated circuits (IC) such as liquid crystal displays (LCDs) and solar cells^1^,^2^,^3^,^4^,^5^,^6^. The oxide semiconductor TFT is a leading candidate for next generation large-area electronic systems, as it can be fabricated at low temperature with high uniformity over large areas, with transparency, high mobility, and high bias stability. Among oxide-based materials, amorphous In–Ga–Zn oxide (a-IGZO) is of particular interest.

Defect density such as sub-gap density of states (DOS) is regarded as one of the most essential material properties for TFTs^7^,^8^,^9^,^10^,^11^,^12^,^13^,^14^,^15^,^16^,^17^. The spectroscopic characterization of this key property has been widely explored, including DC current-voltage (I–V) measurements, capacitance-voltage (C–V) measurements, temperature dependent measurement (DCIV), and optical measurement^1^,^7^,^8^,^9^,^10^,^11^,^12^. However, most of these methods are time-consuming, expensive and lack of accuracy confirmation. The temperature and photoemission dependent measurements require extra and specific instruments for temperature variation measurement or lighting considtions^1^,^7^,^8^,^9^; whereas IV and CV measurements take assumptions and require a lot of mathematical deduction^11^,^12^. For example, the temperature dependence of DOS which has been reported can suggest the benefit of this pure electrical spectroscopy. Previously, the temperature dependency of DOS has been discussed in details^19^,^20^. K. Abe *et al*. showed and discussed about the DOS with different temperature overlapped^19^. It is based on the assumption that the localized DOS, N(E), only falls exponentially with respect to energy E from the conduction band edge to the midgap. That is, N(E) = N_G_exp[(E-Ec)/kT_G_], where N_G_ is the localized state density just under the conduction band edge and T_G_ is the characteristic temperature. This exponential model is employed in some papers^19^,^21^, while slightly different from other publications^7^,^8^. Meanwhile, Y. Liu *et al*. discussed the temperature dependence of DOS based on the measurement of the low frequency (1/f) noise. It shows a noticeable increase in DOS at high temperature^20^. The nice discussions of previous publications suggest that our simple and electrical measurement is needed as a diagnostic tool to qualify the properties of the amorphous semiconductors, which can directly extract the DOS without temperature or optical interference. (This method here, which uses transient pulse signal for transient defect detection, could be similar with the real working situation where most TFTs are working in high frequency conditions instead of DC conditions).

Besides, the measurements under static state conditions could not be used to describe the electrical behavior under the transient working conditions properly. Most of the methods for DOS extraction applied on a-Si TFTs have been applied on amorphous oxide semiconductor TFTs, except transient current spectroscopy without the involvement of temperature or photoemission^13^.

In this manuscript, the transient current spectroscopy (TCS) is upgraded into a dual gate pulse spectroscopy (GPS). By this transient, simple and isothermal dual gate pulse spectroscopy, DOS in amorphous oxide semiconductor TFTs is extracted and modeled. In this way, the intrinsic properties of amorphous oxides and even other semiconductor materials could be characterized in a more pure way.

Our work focuses on the oxide-based TFTs with a-IGZO semiconductor that exhibit room-temperature field effect electron mobility >10 cm^2^/V· s^14^,^15^,^16^. The ratio of In:Ga:Zn of all samples is 1:1:1. The channel lengths of the IGZO TFTs are 10–100 μm, with the width of 50 μm. We measured the transient current for DOS extraction, and the static CV characteristics for surface potential bending, i.e., change of Fermi level in the interface. Based on our results, we have developed a DOS model for the observed transient behavior, which could be applied as simulation tools to characterize the observed electrical behavior.

## Experimental

**Figure 1:**
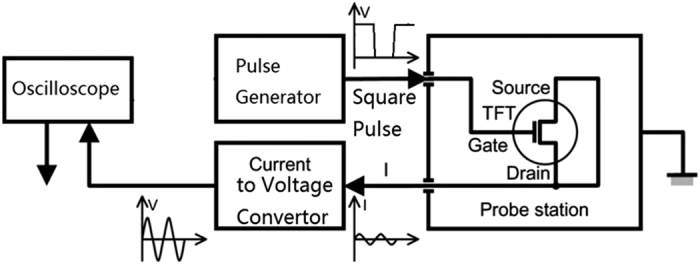
Typical measurement setup for our dual gate pulse spectroscopy. With the permission of Author of ref. 10, Professor Mutsumi Kimura in Department of Electronics and Informatics, Ryukoku University.

Figure 1 shows a block diagram of the apparatus. The pulse generator is Agilent 33500B Waveform Generator. The signal is recorded with Agilent DSO-X 2024A Digital Storage Oscilloscope. TFT sample wafer is measured with the probe station inside an optically and electromagnetically screened box. A train of pulses V_G_ is applied at the gate terminal with fixed rise-time t_r_, fall-time t_f_, pulse amplitude V_T_ and based voltage 0 V. The source and drain terminals are connected to the input of a current-to-voltage converter at a virtual ground potential. A digital storage oscilloscope is used to measure the output signal from the current-to-voltage converter. Signal averaging is employed to stabilize the output signal and improve the signal-to-noise ratio of the signal. The output waveform is averaged 512 times before storage. The amplitude of the gate pulse changed from a top value V_T_ to a base value V_B_ = 0 V, so that the charge stored in the TFT capacitor would flow out of the channel and give a rise to the transient current. The higher V_T_ is, the more electrons from the larger scale of energy bands and thus DOS could be emitted. The measured output includes I_S_ + I_D_, the displacement currents associated with the transistor’s intranodal capacitances; and Istay, the current due to stray capacitances. Istray is measured using the same measurement system, with the probe needles in the same position but detached from the contact pads. The undesired components other than discharging current from DOS emission could be eliminated by measuring two series of gate pulses with two adjacent V_T_ separately.

## Results and discussion

**Figure 2:**
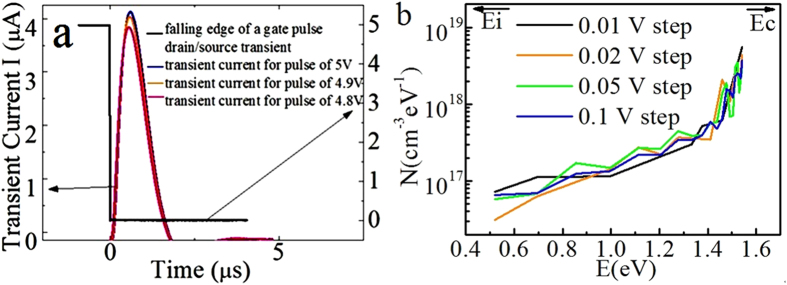
(**a**) Typical transient current versus time curve after a gate pulse falls from V_T_ to 0. (**b**) DOSs extracted from dual gate pulse spectroscopy with different intervals, 0.01, 0.02, 0.05 and 0.1 V, showing good agreement among them.

To obtain the sub-gap DOS distribution of the materials, we need to know both DOS and corresponding energy levels. As shown in Fig. 2a, the measurement result from the IGZO TFT is a typical transient current response. When V_G_ decreases from V_T_ to 0, a transient current I_T_ arises, shows a peak and decreases slowly afterwards.

The TFT sample under test could be regarded as a model of lumped sub-transistors. In the model, the TFT is actually containing two sub-transistors with two symmetric capacitors and two resistors. The channel capacitance and resistance change according to the voltage on gate terminal V_G_. In order to eliminate the parasitic noise effect to an acceptable amount, in the experiments, instead of using a single pulse measurement, we used two gate pulses to obtain two *I*-t curves. It is assumed the system signals introduced by the two pulses such as channel resistance and stray impedances are similar except the DOS, when the difference between 2 consecutive pulses is 0.1 V. This is because that 0.1 V is regarded as small enough to be the typical amplitude of the AC signal for a standard CV measurement^10^. By using 0.1 V difference between two consecutive pulses, the channel carrier increase is regarded as negligible and the system noises are considered to be the same. Only the defects in these two corresponding sub-gap ranges swept by the two gate pulses are different. For CV, +/−50 mV is applied across each step, and therefore this +/−50 mV can be considered as the negligible disturbance. Here we use 0.1 V interval and assume this can be considered also negligible based on the interval comparisons. The extracted DOS using different voltage intervals have been compared, i.e., 0.01, 0.02, 0.05 and 0.1 V. The good agreement as shown in Fig. 2b can be used as a strong support for this assumption.

When we obtain the signal following a gate pulse of a height V_T_, there is a top level in the energy band E_T_ corresponding to V_T_. Here the signal integration from transient *I*-t curve, Q(V_T_), comes from the DOS under E_T_. When we obtain the signal following a gate pulse of a height (V_T_ − 0.1 V), there is a top level in the energy band E_T_’ corresponding to (V_T_ − 0.1 V). Here the signal integration from transient *I*-t curve, Q(V_T_ − 0.1 V), is attributed to electron emission from the DOS under E_T_’. The difference between Q(V_T_) and Q(V_T_ − 0.1 V) is mainly caused by the electron emission from the corresponding DOS at E_T_ within the energy levels where △E_T_ = E_T_ − E_T_’ covers. The value range of V_T_ is within −10 V ~ 10 V. Then the charge emitted from DOS between the dual pulses would give rise to net DOS transient current, which can be written as:


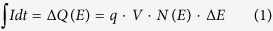


where N(E) is the mean DOS per volume V over energy range ΔE = E_F,VT_ − E_F,VT−0.1_ _V_.

The surface potential is calculated from the CV measurement^10^.


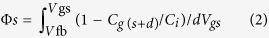


**Figure 3:**
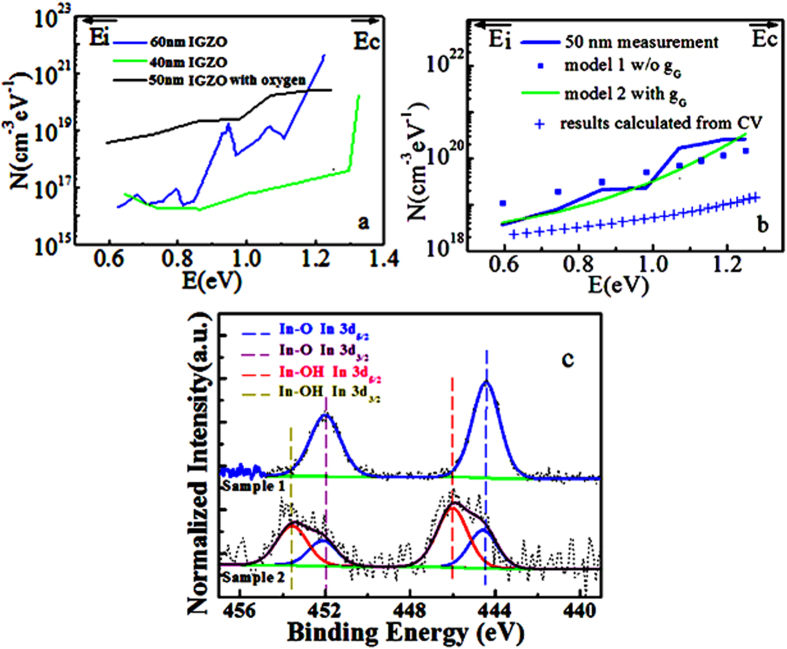
(**a**) DOSs extracted from dual gate pulse spectroscopy. (**b**) DOS from dual gate pulse spectroscopy compare to that extracted from conventional CV method (scatter)^10^ and calculated models with and without *g*_*G*_. (**c**) XPS analysis of 60 nm (sample 1) and 40 nm (sample 2) thick IGZO channel samples fabricated in the same instrument, respectively.

Given the energy band and N, we are able to obtain DOS distribution in energy band gap, as shown in Fig. 3. It is assumed that the energy band gap is about 3 eV. A set of device samples with different channel thickness 40 nm, 50 nm and 60 nm, show similarly increasing trends of DOS distribution. As seen in Fig. 3a, DOS increase from the midgap to the conduction band and a peak near the conduction band edge, which agrees well with the trend reported in previous publications^7^,^8^,^10^,^11^,^12^,^16^. The results of our samples are similar to the published data^2^,^7^,^8^,^10^. The similar order of magnitude and the similar distribution of DOS along the energy band suggest that this measurement is reasonable. What’s more, it is under a transient electrical measurement condition. This might be more similar to a real working condition in the real life, where a TFT is always switched on and off frequently.

The experimental results could be described with the model involving both Gaussian and exponential components, which is also compatible with typical conclusions^7^,^8^. The slight differences are reasonable because different process and composites could lead to different amount of defects and crystal distortion. The transistors with 40 nm, 50 nm and 60 nm channel thicknesses were developed with different oxygen level and time. While there are different processing conditions and wafers, the DOS could be different, which is compatible to the previous publication^10^.

The comparison of this updated dual gate pulse spectroscopy and conventional CV measurement for DOS distribution is given. As shown in Fig. 3b, compared to the conventional method based on calculation from CV measurement, DOS extracted from our dual gate pulse spectroscopy shows a more obvious increasing trend from midgap to conduction band. In Fig. 3b, DOS extracted from CV method is much lower than DOS from the new method. This difference is feasible because CV method is a quasi static measurement, it might not be able to catch completely the transient traps that are stimulated by the gate pulse. The result difference between CV and our method suggest that, the subgap DOS extracted from our method using dual pulses, might be more inclined to include transient traps in the semiconductor energy bandgap.

As shown in the XPS analysis of Fig. 3c, the devices have been fabricated by different instruments, metals and times so that the composition of devices are different. These two samples correspond to IGZO made by the same RF sputtering instruments with different time and thus thickness (i.e., 40 nm to 60 nm). Figure 3c showed the main band of In core levels 3d_5/2_ and 3d_3/2_, the In 3d_5/2_ peak was observed at 444.4 eV, in agreement with the reported value for In-O^22^. The In 3d_5/2_ peaks were observed at 444.4 eV and 445.9 eV, the peak at 445.9 eV could be assigned to In-OH^22^.

XPS measurements were carried out with a high spatial- and energy-resolution XPS Kratos Axis Ultra^DLD^ spectrometer. Figure 3c shows the comparison of sample 1 and sample 2. Both samples were obtained in the same measurement conditions as follows. Initially, the XPS data is obtained. The XPS spectra were calibrated relative to the reference energy value of the carbon 1 *s* core level at 284.8 eV. Afterwards, all XPS spectral peaks were fitted with Vision Processing 2.3.0 Beta Software using Gaussian-Lorentzian (30% Lorentzian) line shapes and the residual background was removed with the help of the Shirley method. As required by theory, In 3d spectral lines consist of two peaks, a spin-orbit doublet, i.e. In 3d_5/2_ and In 3d_3/2_. The In 3d_3/2_ peak was fitted entirely from the fit parameters of the In 3d_5/2_. The In 3d_3/2_ peak intensity was assigned precisely three fifths of the In 3d_5/2_ peak intensity as required by theory. The two peaks were assigned identical FWHM (full width at half maximum of the peak) values. The In 3d_3/2_ peak was separated from the In 3d_5/2_ peak by 7.54 eV and this splitting was used to fit all In 3 d spectra. The XPS spectra have been fitted on the assumption that no chemical shift of the In-O bond in both samples during the sample preparation, and both samples’ In-O In 3d_5/2_ peak have the similar FWHM and peak position as the blue line in Fig. 3c shows, which are typical assumptions for XPS spectra fitting. The peak parameters derived from the best fittings were listed in Table 1. We found another different peak in the fitting of sample 2 from that of sample 1. The different species could be defined by comparing its FWHM (full width at half maximum of the peak) and peak position to the standard handbook of XPS^22^. We identified the different species maybe have In-OH bond.

We could extract the DOS based on the DC measurement also^19^. The average DOS could be calculated from the subtheshold slope of the transfer curves. The results are 3.23 × 10^19^/(cm^3^ · eV) and 4.42 × 10^18^/(cm^3^ · eV) for 50 and 60 nm thick IGZO samples. The reason why the 50-nm sample has a higher average DOS is because its forming process did involve oxygen supply, having more oxygen vacancies^8^. The results are consistent with the numbers of DOS result from this updated dual gate pulse spectroscopy. Such comparison enforces the scientific validity and consistency for the developed novel DOS characterization method.

There are four components in the typical sub-gap DOS modeling of TFTs, including: acceptorlike exponential distribution, acceptorlike Gaussian distribution, donorlike exponential, and donor like Gaussian distribution in the deep states^7^,^8^. Taking into account that the diode sample investigated is operated in an n-type semiconductor and n-channel mode, the acceptor-like states are considered mainly here. We test model 1 with only the acceptor-like exponential DOS described as


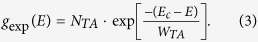


where *N*_*TA*_ is the intercept density at *E*_*C*_, *E*_*C*_ is the conduction band energy, *E* is the state energy, and *W*_*TA*_ is the characteristic decay energy.

Model 2 includes both Eq. (3) and the acceptor-like Gaussian DOS described as


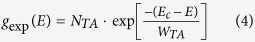


where *E*, *E*_*c*_ and *E*_*0*_ are the trap energy, the conduction band energy and central energy, respectively; *N*_*TA*_, *W*_*TA*_, *N*_*GA*_ and *W*_*TA*_ are the conduction band intercept density, the characteristics energy of the tail states, the density of at *E*_*0*_ of the Gaussian distribution, and the characteristics energy of the Gaussian distribution, respectively. The optimized parameters are summarized in Table 2. *N*_*TA*_, *W*_*TA*_, *N*_*GA*_, and *W*_*GA*_ were determined by the experimental data. Exponential DOSs are used for the tail states near the conduction and valence band edges and Gaussian DOSs are used for the deep gap states, in the case of both hydrogen amorphous silicon(a-Si:H) and amorphous IGZO^7^,^8^. The mobility μ is treated as an average number, 10 cm^2^ · V^−1^ · s^−1^. It is extracted from the measurement of the transfer curve according to the conventional method^19^ and selected to be within a reasonable range according the previous publications^7^,^8^. The detailed parameters and models could be referred to the previous publications^7^,^8^,^21^,^23^.

**Figure 4:**
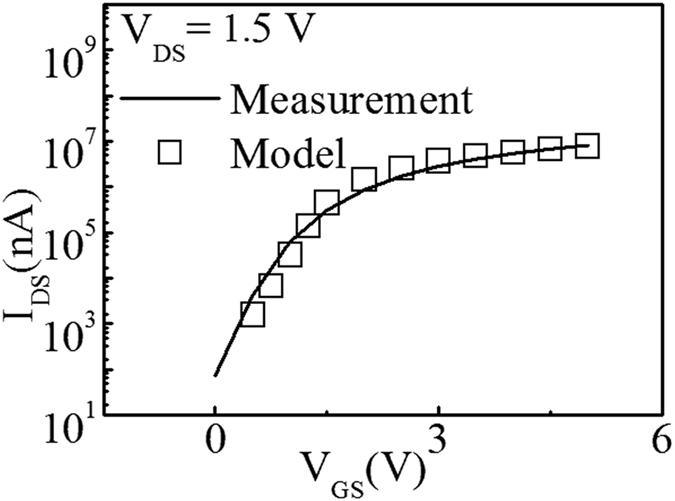
Measurement and simulation using DOS extracted from dual gate pulse spectroscopy, which shows a quite nice fitting between measurement and simulation.

Therefore, we should compare these two models, model I: *g* = *g*_G_, and model II: *g* = *g*_G_ + *g*_exp_. Model 1 considered Gaussian states only, while model 2 incorporated both tail states and deep gap states. The simulations according to these two models are used for the investigation^7^,^8^. As shown in Fig. 4, the simulated data of model II are more similar to the DOS extracted from measured transient current curves. This suggests the parameters in the simulation models could be used to describe the DOS profiles under transient status. The variation in the curve suggests that model II might be more suitable for DOS extracted from transient channel current measurement. The experimental results could be described with the model involving both Gaussian and exponential components, which is also compatible with the typical conclusions^7^,^8^.

In our COMSOL simulation, we selected the reasonable parameters and models. A quite nice fitting is shown between the model and measurement results, as shown in Fig. 4. Therefore, the availability of our measurement for DOS extraction is confirmed by simulation tools^7^,^8^.

Therefore, the validity of this dual gate pulse spectroscopy could be demonstrated. Initially, the DOS distribution extracted using dual gate pulse spectroscopy shows the similar trend with published results. Besides, DOS of our samples extracted by dual gate pulse spectroscopy show similar trend with DOS extracted by conventional methods. What’s more, the experimental results could be described with the model involving both Gaussian and exponential components, which is also compatible with the typical conclusions. Last but not least, the simulation of IV curve supports our measurement results.

## Conclusions

In summary, a key transient electrical property of trap densities, sub-gap DOS of a-IGZO thin films could be measured by a simple and convenient dual gate pulse spectroscopy method, especially this method can tell the DOS difference between different temperatures. Based on this method, a model is proposed to describe sub-gap DOS under transient state measurement. The different samples with difference channel thickness and composites have been investigated, which illustrate a similarly increasing DOS distribution trend along the energy bandgap. The DOS model study might be helpful for better understanding of intrinsic properties of amorphous oxide materials and thus give a hint for typical oxide-based devices control, modeling and improvement. The method attributes the transient current to the sub-gap DOS under the transient bias status. Besides, the method is temperature and optics independent, so it might be used to study the temperature and optical dependence of the DOS distribution, which might be further investigated. We could employ this simple, electrical and convenient method to characterize the material intrinsic property of sub-gap DOS distribution under a transient electrical measurement condition.

## Additional Information

**How to cite this article**: Dai, M. *et al*. A Direct Method to Extract Transient Sub-Gap Density of State (DOS) Based on Dual Gate Pulse Spectroscopy. *Sci. Rep.* **6**, 24096; doi: 10.1038/srep24096 (2016).

## Figures and Tables

**Table 1:** Peak parameters derived by fitting spectra (B.E. Max and FWHM in unit of eV).

Composition	In 3d_5/2_ spectra In-O peak			In 3d_5/2_ spectra In-OH peak		
	B.E. Max	FWHM	% of In 3d_5/2_	B.E. Max	FWHM	% of In 3d_5/2_
Sample 1	444.4	1.58	100			
Sample 2	444.4	1.57	37.3	445.9	1.7	62.7

**Table 2:** Optimized fitting parameters for the IGZO sample with 50 nm thick channel.

	Model I	Model II
*N*_*TA*_(cm^−3^ · eV^−1^)		1.2 × 10^17^
*W*_*TA*_ (eV)		0.5
*N*_*GA*_ (cm^−3^ · eV^−1^)	3 × 10^18^	5 × 10^17^
*W*_*GA*_ (eV)	0.4	0.45
μ(cm^2^·V^−1^ · s^−1^)	10	10
*Eg(eV)*	3.2	3.2

## References

[b1] Kamiya T., Nomura K., Hirano M. & Hosono H. Electronic structure of oxygen deficient amorphous oxide semiconductor a-InGaZnO_4−x_. Appl. Phys. Lett. 89, 203501 (2006).

[b2] Lee S. & Nathan A. Localized tail state distribution in amorphous oxide transistors deduced from low temperature measurements. Appl. Phys. Lett. 101, 113502 (2012).

[b3] Hayashi K. . Electron traps in amorphous In–Ga–Zn–O thin films studied by isothermal capacitance transient spectroscopy. Appl. Phys. Lett. 100, 102106 (2012).

[b4] Yabuta H. . High-mobility thin-film transistor with amorphous InGaZnO_4_ channel fabricated by room temperature rf-magnetron sputtering. Appl. Phys. Lett. 89, 112123 (2006).

[b5] Kang D. . Amorphous gallium indium zinc oxide thin film transistors: Sensitive to oxygen molecules. Appl. Phys. Lett. 90, 192101 (2007).

[b6] Kim M. . High mobility bottom gate InGaZnO thin film transistors with SiO_x_ etch stopper. Appl. Phys. Lett. 90, 212114 (2007).

[b7] Hsieh H. H. . Modeling of amorphous InGaZnO_4_ thin film transistors and their sub-gap density of states. Appl. Phys. Lett. 92, 133503 (2008).

[b8] Fung T.-C. . Two-dimensional numerical simulation of radio frequency sputter amorphous In–Ga–Zn–O thin-film transistors. J. Appl. Phys. 106, 084511 (2009).

[b9] Chung H. J. . Bulk-limited current conduction in amorphous InGaZnO thin films. Electrochemical and Solid-State Letters 11, H51 (2008).

[b10] Kimura M. . Trap densities in amorphous-InGaZnO_4_ thin-film transistors. Appl. Phys. Lett. 92, 133512 (2008).

[b11] Lee S. . Extraction of Sub-gap Density of States in Amorphous InGaZnO Thin-Film Transistors by Using Multifrequency Capacitance-Voltage Characteristics. IEEE Electron. Dev. Let. 31, 231–233 (2010).

[b12] Kim C. E. . Density-of-States Modeling of Solution-Processed InGaZnO Thin-Film Transistors. IEEE Electron. Dev. Let. 31, 1131–1133 (2010).

[b13] Nickel N. . Defect creation in the accumulation layer of a-Si:H thin-film transistors. Philosophical Magazine B. 1990 61, 25.41–261 (1990).

[b14] Ahn S. E. . Metal Oxide Thin Film Phototransistor for Remote Touch Interactive Displays. Advanced Materials. 24, 2631 (2012). 22499356 10.1002/adma.201200293

[b15] Wu Y. . Solution-Processed Hybrid Cathode Interlayer for Inverted Organic Solar Cells. ACS Appl. Mater. & Interfaces 5, 10428–10432 (2013). 24138511 10.1021/am404053e

[b16] Bae H. . Modified Conductance Method for Extraction of Sub-gap Density of States in a-IGZO Thin-Film Transistors. IEEE Elec. Dev. Letters 33, 1138–1140 (2012).

[b17] Dai M. & Ye K. Observation and mechanism explanation of the parasitic charge pumping current. Microelectronics Reliability 50, 1915–1919 (2010).

[b18] Pan T. . Comparison of structural and electrical properties of Lu2O3 and Lu2TiO5 gate dielectrics for a-InGaZnO thin-film transistors. Journal of Applied Physics. 116, 194510 (2014).

[b19] Abe K. . Mobility- and temperature-dependent device model for amorphous In-Ga-Zn-O thin-film transistors. Thin Solid Films 559, 40–43 (2014).

[b20] Liu Y. . Temperature-dependent drain current characteristics and low frequency noises in Indium Zinc Oxide thin film transistors. Chin. Phys. Lett. 32, 088506 (2015).

[b21] Ghittorelli M. . Accurate analytical modeling of amorphous InGaZnO thin-film transistors accounting for trapped and free charges. IEEE Trans. Elec. Dev. 61, 4105 (2014).

[b22] XPS Chemical Bonds (Date of access: 08/12/2015) http://www.lasurface.com/database/liaisonxps.php (2015)

[b23] Dai M. & Dai N. Logic circuit function realization by one transistor. Nano Letters 11, 5954–5956 (2012). 23075033 10.1021/nl303386b

