# Corrigendum: A Direct Method to Extract Transient Sub-Gap Density of State (DOS) Based on Dual Gate Pulse Spectroscopy

**DOI:** 10.1038/srep35519

**Published:** 2016-11-02

**Authors:** Mingzhi Dai, Karim Khan, Shengnan Zhang, Kemin Jiang, Xingye Zhang, Weiliang Wang, Lingyan Liang, Hongtao Cao, Pengjun Wang, Peng Wang, Lijing Miao, Haiming Qin, Jun Jiang, Lixin Xue, Junhao Chu

Scientific Reports 6: Article number: 2409610.1038/srep24096; published online: 06 14 2016; updated: 11 02 2016.

In this article, the Acknowledgements section is incomplete:

‘The authors appreciate the help of Professor Arokia Nathan and Dr. Sungsik Lee in University of Cambridge and Professor Mutsumi Kimura in Department of Electronics and Informatics, Ryukoku University. This work was supported by Research Exchanges with India and China scheme, Royal Academy of Engineering, UK, National Natural Science Foundation of China (Grant No. 61106090), the Spring Project in Ningbo Institute of Material Technology and Engineering, the Ningbo Natural Science Foundation of China (Grant No. Y10814VA08, No. 2014B82004), and Youth Innovation Promotion Association, Chinese Academy of Sciences. Professor Mutsumi Kimura from Department of Electronics and Informatics, Ryukoku University is appreciated for his explanation of CV measurement and calculation for DOS’.

Should read:

‘The authors appreciate the help of Professor Mutsumi Kimura in Department of Electronics and Informatics, Ryukoku University. This work was supported by the National Natural Science Foundation of China (Grant Nos 61106090, 61574147, 61274132), Ningbo Municipal Natural Science Foundation (No. 2014A610011), the Ningbo Natural Science Foundation of China (Grant Nos 2015A610034, 2011A610110, No. 2014B82004), the State Key Basic Research Program of China (2013CB922300), Youth Innovation Promotion Association, Chinese Academy of Sciences and Royal Academy of Engineering, UK. The author appreciates the K.C. Wong Magna Fund in Ningbo University, China’.

